# Supplementary material for: Are carbon emissions trading and green financial instruments synergistic? -Comprehensive quantitative research based on content analysis
Source: PLoS One. 2024 Mar 7;19(3):e0298601. doi: 10.1371/journal.pone.0298601 (PMC10919601; doi:10.1371/journal.pone.0298601)
Supplement: S1 Data — (ZIP) [file pone.0298601.s001.zip › Data/地级市Z_F工作报告.zip!/╣π╬≈/│τ╫≤╩╨/└┤╫╘╣1⁄2╓┌║┼í╛┬φ┐╦╩2╛▌═°í┐_2015.docx]

各位代表：

现在，我代表市人民政府向大会作政府工作报告，请予审议。同时，请市政协委员和列席会议的同志提出意见。

一、2014年工作回顾

过去的一年，是全面贯彻落实党的十八届三中全会精神、全面深化改革的开局之年。面对复杂多变的经济形势和艰巨繁重的改革发展稳定任务，全市各族人民在自治区党委、政府和市委的坚强领导下，紧紧围绕全面深化改革、实现“两加”“两成”的目标任务，以开展党的群众路线教育实践活动为动力，扎实推进“稳增长、促改革、调结构、惠民生”各项工作，大力实施“七个促增”，较好完成了市三届人大五次会议确定的各项目标任务，实现了经济社会平稳持续健康发展。

——经济增长总体平稳。初步核算，全年生产总值649.72亿元，比上年增长8.3%，增速排全区第八位，保持在合理增长区间。财政收入73.16亿元，比上年略有增长，总量保持全区第9位；财政质量进一步优化。新常态下，我市经济运行总体平稳、稳中有进、稳中向好、稳中提质。

——三次产业协调发展。农业生产稳步增长，规模以上工业增加值226.33亿元，增长11.5%，增速高于全区水平，三产占GDP比重提高2.6个百分点。三次产业结构呈现积极变化，由上年的25.6:42.4:32.0优化调整为22.7:42.7:34.6。

——投资拉动持续有力。固定资产投资达到548.64亿元，增长21.7%，增速高于全区5个百分点，位居全区第一。重大项目拉动投资增长明显，全市在建5000万元以上重大项目404个，全年完成投资258亿元，占投资总额47%。

——对外贸易增势强劲。外贸进出口持续快速增长，全年完成146.94亿美元，增长43%，增速分别高于全国、全区平均水平39.6个百分点和19.5个百分点。进出口总额和出口总额继续稳居全区首位，占全区比重达三分之一和二分之一以上；边境小额贸易进出口总额跃居全国首位，成为全国边境贸易第一大市。

——民生改善切实加强。全年民生支出占一般公共预算支出比重提高到75.4%，同比提高3.3个百分点。人民生活水平不断提高，城镇居民人均可支配收入23184元，增长8.9%，农民人均纯收入7707元，增长8.9%，均高于GDP增速。物价总体平稳，全年CPI累计上涨2.4%，低于自治区下达的调控目标。城乡消费市场持续活跃，社会消费品零售总额108.44亿元，增长12.5%。

这些成绩，标志着我市又向“两加”“两成”目标迈出了坚实的一步，必将激励我们在新的征程上开拓进取、奋勇前进！

一年来，我们主要抓了以下八个方面工作。

（一）全面深化改革，经济发展注入新活力。明确了36条146项改革任务，重点推进7个方面的改革工作。市县政府职能转变和机构改革扎实推进，取消、下放197项行政审批事项，审批项目减少17.6%，53个部门公布行政审批事项清单。市公共资源交易中心建成运行。工商登记制度改革成效明显，新登记公司数量和注册资本分别增长87.6%和210.2%。事业单位分类制度改革有序开展。乡镇机构改革全面推进，全市所有乡镇、街道建立了政务服务中心，超过50%的村、社区设置了便民服务站。在全区率先开展农村土地承包经营权确权登记颁证试点工作，完成确权5.96万亩，“小块并大块” 累计完成92.4万亩；在龙州成立广西边境市县首家综合性农村产权交易所。华侨农林场体制改革方案通过自治区政府审批。沿边金融综合改革取得新成果，启动运行中国（广西凭祥试验区）东盟货币服务平台，开展经常账户下个人跨境人民币结算业务试点。成立凭祥重点开发开放试验区管委会和水口、爱店、硕龙口岸经济区管委会，积极推进边民互市贸易改革试点工作。进一步理顺市与城区权责关系，继续完善城市工业区管理体制，实现“园区事务在园区内办结”。

（二）加力开放合作，开放型经济水平不断提升。充分发挥北部湾经济区、珠江—西江经济带和左右江革命老区“三区叠加”的政策优势，大力实施“双核驱动、三区统筹”战略，全方位扩大开放合作。积极搭建开放合作新平台，加快南宁—崇左—凭祥对外开放经济带建设。凭祥重点开发开放试验区、中越凭祥—同登跨境经济合作区、龙州边境经济合作区申报和建设有新进展，凭祥综合保税区保税业务值居全国同类边境保税区首位。友城工作再有新成效，与美国弗农山市、墨西哥特拉维利尔潘市、法国萨姆瓦纳市签署国际友好城市关系意向书。宁明爱店口岸获批升格为国家一类口岸。推行凭祥口岸关检合作“三个一”通关模式改革，申报项目从169项优化为92项，企业办理查验等候时间减少50%以上。外贸企业迅猛发展，全市7家企业进入广西外贸出口额前10强。成功举办中国—东盟家居业知名企业崇左行活动和第四届陆路东盟国际商务文化节。全年完成区外招商引资到位资金285.58亿元，其中全口径实际利用外资1.62亿美元。

（三）加快南崇经济带建设，产业结构转型优化。实施工业跨越发展“1915工程”和“十大行动计划”，打好产业转型升级攻坚战，深化拓展产业发展空间。园区建设加快推进，中泰（崇左）产业园、广西·东盟特色食品产业园发展规划编制完成。全年园区工业总产值420亿元，增长13.9%，占全市工业总产值的71.9%。“糖矿红”支柱特色产业提升发展。糖业方面，积极引导制糖企业发展循环经济，拉长产业链，湘桂糖业酵母抽提物等一批循环经济项目竣工投产，8家制糖企业被评为广西工业循环经济先进企业。矿业方面，在提升发展锰业的基础上，加快开发利用稀土、铜、铝土、铁矿等资源，中信大锰锂离子动力电池等循环经济项目启动建设，南国铜业项目建设顺利推进，中铝稀土分离生产线项目实现试产，中信大锰大新分公司电解金属锰等一批项目竣工投产。红木产业方面，红木加工基地一期实现竣工，二期26家企业开工建设；红木品牌培育取得新成效，全市新增4个广西著名商标、5家广西名牌产品企业。新兴产业方面，大力发展林产家具、边贸加工等产业，龙赞林业循环经济产业园项目落户崇左工业区，7个坚果加工项目实现开竣工，新的增长点逐步形成。企业实力不断增强，全年营业收入超亿元企业达83家。非公经济快速发展，非公工业对工业增长贡献率达73.9%。全面完成年度淘汰落后产能任务，单位生产总值能耗下降1.7%。大力发展现代农业，落实“双高”糖料蔗示范基地30万亩，超额完成自治区下达任务，扶绥“甜蜜之光”现代特色农业（核心）示范区即将通过自治区验收，甘蔗综合机械化水平全区第一，庭院特色养殖走在全区前列。

（四）狠抓投资和项目建设，发展后劲持续增强。落实市四家班子联系项目建设责任制，抓好项目建设，促进投资快速增长。市级层面以上统筹推进的207个重大项目完成投资143.5亿元，其中35个自治区层面重大项目完成投资48.6亿元。崇左电厂等项目前期工作加快推进，驮英水库及灌区工程项目建议书已获国家发改委批复，南方水泥、红狮水泥项目即将点火试产。湘桂铁路南宁至凭祥段扩能改造项目列入自治区“市市通高铁”计划，崇左至靖西高速公路崇左段累计完成57.4%投资量，农村公路通达率提高到87.4%，左江Ⅲ级航道疏浚工程主体完工。财政支持项目建设力度不断加大，安排1.46亿元支持工业园区基础设施建设，投入城乡建设资金11.47亿元。金融信贷运行平稳，全年存贷款余额分别增长12%、14%，增速分别排在全区第4位、第7位。加快留邕资产盘活。积极争取金融支持，市本级融资平台获批贷款近百亿元，已提款21.6亿元，为城市建设和产业发展提供了强有力的资金支持。

（五）加快新型城镇化建设，城乡面貌明显改观。全市城镇化率预计达36.5%。中心城市建设步伐加快，“一江一园一改一桥两校三中心八路”项目进展顺利，左江崇左城区河段防洪综合治理工程前期工作积极开展；园博园基础设施项目建设有序推进；棚户区改造工程获得国开行贷款72.27亿元；崇左大桥启动建设；市体育中心、城市规划建设展示馆、水口湖整治工程等项目加快建设。城区道路、供水管网、城镇污水生活垃圾处理设施等日趋完善，荣获“广西园林城市”称号。城镇建设扎实推进，完成310个村镇规划编制，保障性住房新开工9679套、基本建成4085套，小城镇建设“书记工程”、名镇名村建设、城乡风貌改造、农村危旧房改造共完成投资9.98亿元。深入开展“美丽广西·清洁乡村”活动，扎实推进生态乡村建设，农村环境综合整治取得实效，城乡人居环境日益改善。

（六）大力发展文化旅游，崇左知名度日益提升。大力实施“发现山水崇左·圆梦别样桂林”旅游发展战略，深入推进旅游“25671”工程，大德天景区升级改造、龙谷湾旅游休闲度假区等项目加快建设。左江花山岩画文化景观申报世界文化遗产工作取得重大突破，被确定为2016年中国唯一申报项目。德天瀑布被确定为广西2015年创5A重点景区，凭祥红木文博城被评为4A级景区。大新县、凭祥市积极开展广西特色旅游名县创建工作，宁明县、龙州县列入广西特色旅游名县创建备选县。“中国白头叶猴之乡”、“三月三”民俗活动等文化旅游品牌全面打响。全年接待游客和旅游收入分别增长15%和22.3%。深入推进生态建设，全面完成主要污染物减排任务，环境质量总体保持良好。荣获“国家珍贵树种培育示范市”和“中国木棉之乡”称号，龙州左江河段和大新黑水河两处湿地被列为国家级湿地公园，森林覆盖率预计提高到54.6%。

（七）统筹发展社会事业，社会大局和谐稳定。投入资金43.26亿元，全面完成10项为民办实事工程。优先发展教育事业，实施教育“双百工程”，落实6.89亿元建设563个教育项目，城南小学、市机关保育院于9月初开学；九年义务教育巩固率和高中阶段教育毛入学率分别提高到82%、77%。实施精准扶贫，减少贫困人口6万人。实施农村人饮工程，解决11万农村人口饮水不安全问题。扎实推进科技创新，每万人口发明专利拥有量增长125%。繁荣发展文化事业，壮族霜降节列入国家级非物质文化遗产名录，108个村级公共服务中心全面建成。加快发展卫生事业，开展新农合大病保险试点，新农合参合率99.51%，位居全区第二；市第二人民医院揭牌运营，市儿童医院、市妇幼保健院开工建设。稳妥、有效实施单独两孩政策，全面落实诚信计生，人口自然增长率控制在6.25‰。稳定和扩大就业，城镇新增就业1.8万人，城镇登记失业率2.4%，低于自治区控制数1.8个百分点。社会保障水平进一步提高，城乡居民基本养老保险参保率97%、待遇发放率100%；城乡低保“应保尽保”，发放保障金4.5亿元。深入开展民族团结进步创建活动，2个集体和4名个人荣获第六届全国民族团结进步模范称号。国防教育和后备力量建设深入开展，双拥共建工作取得新成绩。食品药品安全监管能力明显提升。安全生产、应急管理、社区纠正、矛盾纠纷排查调处等工作进一步加强。连续11年实现全国“两会”等敏感节点和重大活动期间“零上访、零非访”工作目标，平安建设工作获全区目标管理一等奖，社会公众安全感和社会稳定动态七项工作总分排名继续位居全区前列。

（八）进一步转变作风，发展环境不断优化。深入开展党的群众路线教育实践活动，严格落实中央“八项规定”，严格落实“驻村蹲点”制度，教育实践活动取得积极成果。市政府党组22项立行立改工作全部整改完成，政府部门各项整改工作扎实推进，政府机关干部作风明显转变。预计全市“三公”经费下降13.3%，会议费下降28.9%，培训费下降25.3%。继续开展电视问政直播活动，自觉接受群众监督，积极回应群众关切。全年共办理人大代表建议71件、政协提案107件。法治政府建设稳步推进，廉政建设扎实开展，行政监察和审计监督更加规范，政风、行风建设进一步加强。2014年我市年度投资环境总体满意度排全区第3位，其中投资软环境满意度排全区第2位。

一年来，我市统计、法制、水库移民、国有资产监督管理、质量技术监督、新闻广电、体育、供销合作、二轻、宗教、涉台事务、机关事务管理、接待、驻邕管理、驻京联络、地震、人防、边防、税务、无线电管理、气象、水文、消防、海关、海事、检验检疫、档案、保密、国家安全、老龄、红十字会、地方志等工作取得新进展。供电、保险、邮政、通信、石油、烟草、盐业等行业和工会、共青团、妇联、工商联、科协、文联、侨联、残联、社科联等群团组织在经济社会发展中发挥了重要作用，作出了积极贡献。

回顾过去一年的工作，我们深切体会到，要保持我市经济社会平稳持续健康发展，必须坚持深化改革，扩大开放，紧紧围绕“两加”“两成”，不断激发全市人民的热情和活力推动发展；必须坚持超前谋划、精准施策，狠抓“七个促增”，不断凝聚全市人民的智慧和力量推动发展；必须坚持勇于担当、奋发有为、主动作为，不断增强干部执行力推动发展；必须坚持绿色发展、环境优先，牢固树立“绿水青山就是金山银山”的理念，不断提升绿色生态优势推动发展；必须坚持走群众路线，密切联系群众，持续改进干部作风，不断汇聚赶超跨越的正能量推动发展；必须坚持以民为本，民生至上，经济越困难，越要关注和解决民生问题，让发展成果更多惠及民众，不断夯实基础增强合力推动发展。

各位代表，过去的一年，我们在经济下行压力大、持续时间长的严峻形势下，爬坡过坎、加力加劲、共克时艰，努力开创了加快实现“两加”“两成”的新局面。这是自治区党委、政府和市委统揽全局、正确领导的结果，是市人大及其常委会依法监督和市政协民主监督、鼎力支持的结果，是全市上下同心同德、拼搏进取的结果。在此，我代表市人民政府，向付出辛勤劳动的全市广大干部群众，向各位人大代表、政协委员，向各民主党派、工商联、人民团体、中直区直驻崇左各单位，向驻崇左人民解放军、武警部队官兵，向所有关心支持崇左建设的各界人士，表示崇高的敬意和衷心的感谢！

回顾过去一年，我们也清醒地看到，我市发展中仍存在不少困难和问题。一是经济发展结构性矛盾突出，糖锰产业经营困难，消费需求增长动力偏弱，经济下行压力仍然较大；二是涉及人民切身利益的教育就业、社会保障、医疗卫生等，依然存在诸多不满意的地方；三是政府职能转变仍需加快，各项改革措施有待进一步落地。对此，我们一定要高度重视，采取切实有效措施，破解难题，加快发展。

二、2015年工作安排

今年是全面深化改革的关键之年，是全面推进崇左法治建设的开局之年，是全面完成“十二五”规划的收官之年。当前，我国经济发展进入新常态，资源环境约束条件增多，经济下行压力仍然很大，我市和全国全区一样，经济正处在难熬的转型“阵痛期”，改革发展任重道远。但总的来看，我市仍处于大有可为的重要战略机遇期。特别是“三区叠加”政策效应不断扩大，发展潜力逐步显现，将给今后发展提供更强大的动力。我们要适应新常态，抢抓新机遇，全面深化改革，扩大开发开放，走出一条符合崇左实际、具有崇左特色、体现人与自然和谐发展的新路子。

2015年政府工作的总体要求是：深入贯彻落实党的十八大、十八届三中、四中全会和习近平总书记系列重要讲话以及自治区党委十届五次全会、市委三届五次全会精神，适应新常态稳中求进，奋力推进“两加”“两成”工作，促进全市经济平稳健康发展、社会和谐稳定。

2015年政府工作的主要预期目标是：地区生产总值增长9%，财政收入增长6%，规模以上工业增加值增长12%，固定资产投资增长17%，外贸进出口总额增长16%，社会消费品零售总额增长12%，城镇居民人均可支配收入增长8.5%以上，农民人均纯收入增长10%，居民消费价格指数涨幅控制在3.5%以内，城镇化率38%，城镇登记失业率控制在4.2%以内，城镇新增就业1.6万人，人口自然增长率控制在8.5‰以内。

制定上述目标，主要是从适应新常态、实现“十二五”目标和全面实现小康社会的大背景出发，把握“努力实现一个高于全区平均水平的发展速度”的原则，充分考虑了我市当前发展形势及经济运行情况等综合因素，为我市进一步转方式、调结构，提高发展质量和效益留出空间，总体上实现“调速不减质、量增质更优”。为此，今年重点抓好以下工作。

（一）以问题为导向，持续推进全面深化改革。

深化行政审批制度改革。进一步简政放权，做好“接、放、管”，最大限度减少审批环节和审批事项。规范行政审批管理，优化审批流程，提高审批效率。加强各级政务服务中心建设，加快电子政务建设。全面实施工商登记“先照后证”。进一步规范完善市公共资源交易中心建设，加强公共资源交易监督管理。

深化投融资体制改革。研究提出我市政府核准投资项目目录，尽可能缩小核准范围，减少、简化、整合前置审批。加强政府性债务管理，规范发展政府投融资平台。扩大民间资本市场准入范围，探索PPP模式，鼓励社会资本参与项目投资建设。

深化财税金融体制改革。全面贯彻落实新的《预算法》，深化财政预算管理改革，加强财政收支管理，努力提高财政收入质量，进一步推进预算公开，推广政府购买服务。加强结余结转资金管理，加大重点项目和民生领域支出。支持市城投公司发行企业债券。推进“营改增”等税收制度改革。多渠道扶持中小企业发展，力争小额贷款公司、担保公司实现县域全覆盖。进一步完善金融体系，鼓励各类股权投资基金在崇左聚集，支持域外金融机构在我市设立分支机构。积极参与沿边金融综合改革试验区建设，大力推进跨境人民币结算业务，推进人民币与越南盾特许兑换试点工作。

充分激发非公有制经济活力。鼓励、支持、引导非公有制经济发展，发挥市场在资源配置中的决定性作用，保证各种所有制经济依法平等使用生产要素，进一步激发非公有制经济的发展活力和生产力。大力扶持小微企业发展，积极引导创业投资基金投资小微企业。

盘活资源统筹发展。树立全市资源开发一盘棋的理念，盘活优势资源，促使各类资源要素从分散走向融合。探索坚果加工、林产加工等资源产业发展之路，统筹全市政策，形成统一市场，培育两个新的百亿元产业，形成新常态下新的增长点。

继续推进农村、园区等相关领域体制机制改革。深化农村改革，积极引导农村土地经营权有序流转。加快土地确权登记颁证工作，在14个乡镇全面铺开。加快园区管理体制、华侨农林场体制等改革步伐，统筹推进各领域改革。加快社会信用体系建设。

（二）以“三驾马车”为动力，促进经济平稳增长。

加大投资和项目建设。发挥投资对经济增长和结构调整的关键性作用，推动项目建设。今年市层面统筹推进重大项目204个，年度投资200.2亿元。继续推行市四家班子联系项目建设责任制，强化项目协调服务。市本级财政预算安排5000万元经费，保障重大项目前期工作。推进新开工项目尽快开工，续建项目早日竣工。重点做好生产性项目服务保障工作，依法开展征地拆迁安置工作。加大土地盘活、清理力度，统筹安排产业、民生等重点项目用地指标。多渠道筹措项目资金，力争获得更多中央预算内投资。加大招商引资力度，力争全市区外招商引资到位资金255亿元以上。

努力扩大消费需求。保持物价水平总体稳定。激发消费热情，提升消费能力，努力把更多的住房、汽车、旅游、商贸等消费留在崇左。

提速发展现代服务业。改造提升交通运输、住宿餐饮等传统服务业。大力发展信息、保险等生产性服务业。加快发展物流、科技服务、健康养老、教育、旅游休闲、会展、家政等现代服务业。落实“电商广西、电商东盟”工程，积极发展电子商务、第三方物流、连锁经营等现代流通业态，争取开展跨境电子商务示范创建活动。优化服务业发展环境，整顿和规范市场秩序，出台相关优惠政策，让更多法人商贸企业落户崇左，支持推动一批商贸企业“个转企”、“小上限”。加快发展各类专业市场，推进中国—东盟特色产品大市场、中国—东盟（凭祥）汽摩配专业市场等项目建设，力争市农副产品交易中心今年9月底建成使用，加快在中心城区规划布局汽车及配件、家具、家电、建材等大型交易市场。

提高出口贡献率。支持本地企业扩大自营产品出口。巩固越南传统市场，拓展泰老柬等东盟市场，扩大传统产品出口，积极引导和鼓励企业出口高新技术产品，进一步提高出口贡献率。

（三）以重大基础设施为支撑，不断夯实产业发展基础。

公路方面。推动凭祥至河内高速公路、友谊关货运专用通道等跨境交通项目建设，争取水口至驮隆中越界河公路二桥开工建设。加快建设崇左至靖西高速公路，争取开工建设崇左至水口高速公路，推进隆安至硕龙高速公路前期工作。加快建设德保至天等、驮卢至大新等二级公路，力争开工建设南宁大塘至扶绥渠黎、大新桃城至龙州金龙等二级公路。提升改造农村公路，所有未通硬化路的建制村要在年内全部开工建设，实现95%以上建制村通沥青（水泥）路。

铁路方面。加快湘桂铁路南宁至凭祥段扩能改造项目前期工作，争取2016年开工建设，使崇左早日进入高铁时代，及早优化布局高铁沿线产业，打造高铁经济带。力争防城港经崇左至百色铁路项目列入国家“十三五”规划。

水运水利方面。加快建设左江Ⅲ级航道工程，开工建设崇左港濑湍、新环作业区，力争建成将军岭作业区一期。继续推进驮英水库及灌区项目前期工作，力争年内开工建设。加快推进黑水河灌区项目前期工作。抓好中小河流治理、病险水库除险加固、中央财政小型农田水利重点县等工程建设。

机场建设方面。启动大新德天等通用机场项目前期工作，推动项目早日开工建设。

能源方面。加快崇左电厂项目前期工作，力争年内开工建设。加快发展锰电结合，鼓励锰企业建设动力车间。积极争取将崇左锰业用电纳入百色区域电网，争取中信大锰、海螺水泥等企业纳入自治区第一批直购电试点范围。

（四）以“三区叠加”为新机遇，加快南宁—崇左—凭祥对外开放经济带建设。

积极融入北部湾经济区。着力抓好基础设施、产业升级、商贸物流等项目建设，优化空间布局，促进产业集聚发展。加快实现与“南北钦防”四市各项同城化改革逐步对接。

全力参与西江经济带建设。实施珠江—西江经济带发展规划，加快西江经济带基础设施建设大会战重大项目建设，提升左江航运能力。谋划左江沿江产业布局，加快承接东部产业转移，加速形成沿江产业集群。

全面振兴崇左革命老区。抢抓国家即将出台《左右江革命老区振兴规划》的机遇，大力实施左右江革命老区重大工程建设攻坚3年行动计划。重点实施好重大基础设施、特色产业发展、生态经济、扶贫攻坚、惠民富民“五大工程”，加快崇左革命老区振兴发展。

搭建开放合作平台。积极参与“一带一路”建设。争取凭祥重点开发开放试验区上半年获得国家批复，争取自治区支持东兴试验区的政策全覆盖凭祥试验区。推动中越凭祥—同登跨境经济合作区尽快获得两国政府批准建设。把握泰国为今年中国—东盟博览会主题国的契机，努力推动中泰（崇左）产业园上升到国家层面。加快推进龙州边境经济合作区申报工作。加快凭祥综合保税区二期建设，配合做好保税区管理体制改革。继续深化区域合作，主动对接东盟、欧美国家及港澳台地区，开展经贸交流合作，开辟国际大市场。

大力发展口岸经济。加强和完善口岸和互市点基础设施建设，加快凭祥边境贸易货物监管中心等项目建设，力争宁明爱店口岸验货场和联检楼改扩建工程建成运行。继续推进硕龙口岸升格和友谊关、水口口岸扩大开放，争取在叫隘、那花、布局、岩应等边民互市点开展边境小额贸易。继续推行“5+2”工作制和“24小时预约通关”服务，提升口岸服务功能。加快凭祥口岸关检合作“三个一”改革工作，探索推进“两国一检”工作。继续实施加工贸易倍增计划，推动我市通道经济向口岸经济加快转型。

（五）以转方式调结构为主线，加快推动工农业转型升级。

大力推进工业转型升级。千方百计稳住糖锰传统支柱产业，实施“二次创业”。加快发展蔗糖深加工，谋划发展甘蔗浓缩汁、糖蜜制药、蔗渣生产食用纤维等糖关联产业。改造提升生态型锰精深加工，加快布东生态锰产业园等项目前期工作，推进中信大锰锂离子动力电池、新振锰业中低碳锰铁合金等项目建设，确保沙钢锰业硅锰合金等项目竣工投产。做大做强红木文化产业，加快红木加工基地和红木商品交易中心建设，推进红木与文化元素相融合，打造红木文化品牌。培育发展新产业。加快优质资源开发，将资源优势转变为产业优势。稀土产业方面，推动中铝稀土分离生产线项目尽快达产，力争金石彭源和索维尔项目开工建设，尽快形成高附加值的稀土材料深加工园区。铜业方面，加快南国铜业项目建设，力争年内一期工程竣工投产。铝业方面，加快龙州氧化铝项目前期工作，力争上半年开工，2016年竣工投产。建材业方面，支持海螺、南方、红狮、东泥等重点水泥企业做大做强，鼓励发展膨润土、碳酸钙深加工。边贸加工业方面，实现崇左—东盟农产品采购分拨中心正常运营，加快坚果加工项目和凭祥农产品加工物流园建设。林产业方面，推进龙赞林业循环经济产业园项目建设，提升发展广西剑麻—林产循环科技产业园，加快形成林产家具业加工基地。加快打造特色园区，推进崇左工业区建设，重点培育发展农林产品加工业和稀土产业，用五年左右时间打造成为全区规模较大、特色鲜明的重点产业园区。推进崇左工业区与中心城区、扶绥空港经济区与扶绥县城互动发展。

大力推进创新驱动。鼓励和支持企业进行技术产品创新。继续推进“质量兴市”工作，培育一批名牌产品。加大知识产权保护力度，加强科学技术普及。加快推进科技企业孵化器建设工作。

稳定粮食和甘蔗生产。坚定不移实行最严格的耕地保护和节约集约用地制度。确保全市粮食播种面积稳定在181万亩、粮食总产量50万吨以上。加强粮食仓储设施建设，加大市县中心粮库投入力度。稳定甘蔗种植面积在400万亩以上。继续强力推进“双高”糖料蔗基地建设，确保完成年度31万亩建设任务，进一步提升蔗糖产业质量效益和抗风险能力。

大力发展特色效益农业。加快现代特色农业（核心）示范区建设，每个县（市、区）建设一个示范区，引进一批科技含量高、规模大、带动能力强的龙头企业参与建设。抓好 “菜篮子”工程， 发展热带水果、中药材等特色种植，抓好良种繁育基地建设，发展特色养殖和休闲生态观光农业，打造更多“一乡一品”、“一村一品”特色品牌。

加快构建新型农业经营体系。引导和推动“小块并大块”耕地整治工作，力争今年新增70万亩。积极发展多种形式适度规模经营，新增70个农民专业合作社和一批专业大户、家庭农场、农村专业技术协会。做好农业政策性保险工作。力争全市耕种收综合机械化水平达到49%以上。加大农资市场监管力度。健全农产品流通信息服务体系，加强产销对接，提高农产品流通率。

（六）以城乡一体化为落脚点，加快新型城镇化建设。

进一步加快中心城市建设。加强城市规划管理，加快重点规划编制工作。高质量办好第七届广西园林园艺博览会。以园博会为契机，加快石景林路、环城西路等路网建设，完善中心城市水、电、路、通信、市场等基础设施，加大旧城区基础设施投入力度，提升城市服务功能，增强中心城市辐射带动力。加快左江崇左城区河段防洪综合治理工程建设，启动江南片区沿江路网、防洪堤等整治工作。加快市体育中心、崇左大桥等项目建设，推进崇左三桥扩建工程。力争丽江水厂二期、江南污水处理厂二期等项目竣工使用。

提质扩容发展县城和重点城镇。加快推进南宁—扶绥一体化、凭祥城乡一体化建设，不断扩大城镇规模。加快推进棚户区改造工程、城乡风貌改造和名镇名村建设。加大城镇基础设施建设，今年新开工建设13664套保障性安居工程，对11000户农村危房和6516户城乡房屋外立面进行改造。加强城市地下管网建设改造和规范治理。推进城镇垃圾分类收集处理，城镇污水处理率和生活垃圾无害化处理率达到85%以上。加快推进县县通天然气工程，力争实现城镇燃气普及率92%以上。抓好宽带网络优化提速工程，推进三网融合。

提高城镇管理水平。持续推进城区环境卫生、粉尘污染、交通综合等专项整治，建立长效机制。强化“两违”综合整治，落实“门前三包”，改善市容市貌。我们将努力让文明整洁、靓丽宜居成为崇左市容“新常态”！

（七）以绿色生态为依托，大力发展文化旅游产业。

全力推进花山岩画申遗。加快花山岩画博物馆、花山岩画文化景观展示中心和宁明花山岩画监测站项目建设，扎实做好遗产区环境整治和基础配套等工作，确保2016年申遗成功。以花山申遗为契机，科学谋划和发展左江生态休闲旅游，实现有序开发。

全面打响旅游品牌。实施“发现山水崇左·圆梦别样桂林”战略，积极融入广西旅游“三区一带”新格局，加大旅游资源整合开发力度，推动中越国际旅游合作区建设，引领广西边关风情旅游新发展。继续抓好特色旅游名县名镇创建工作。积极打造长寿养生旅游目的地，争取纳入广西养老服务综合改革实验区，加快中国（崇左）乐养城、石景林景区提升改造和五星级万景健康城综合开发等项目建设，推进大新安平国际旅游度假区等项目前期工作，力争龙谷湾旅游休闲度假区今年10月实现开园，争取德天瀑布景区年内创5A成功。着力解决旅游“最后一公里”、“最后一步路”问题，提升旅游服务水平。办好第二届“三月三祭壮祖赶歌坡赏木棉”活动月等节庆活动，进一步打造民族文化品牌。

深化“美丽崇左”建设。落实生态环境保护责任制，加强基层环境监管能力建设，严格环境保护监管、执法，划定生态保护红线。依法淘汰落后产能，进一步控制污染物排放，全面完成“十二五”节能减排任务。深入推进“美丽广西·生态乡村”活动。扎实推进国家森林城市创建工作，力争森林覆盖率达55%以上。

（八）以改善民生为根本，统筹推进社会事业发展。

继续优先发展教育。加强思想道德建设和精神文明创建工作。加快实施教育“双百工程”，完善教师激励机制，推进义务教育标准化建设，改造和新建一批学校。大力发展学前教育，创建2所以上区级示范园。继续推进普通高中建设工作，扩大招生规模，提高办学质量。推进县级职业教育综合改革，加快国门职业教育园区二期建设。支持广西民族师范学院做好迎接教育部本科教学水平评估工作。

深入实施精准扶贫。认真落实“四到县”制度，扎实推进“两大会战、四大攻坚战”，加大扶贫工作力度，力争实现脱贫6万人以上，全面完成“十二五”贫困村整村推进各项目标任务。进一步落实“领导挂点、单位包村、干部包户”扶贫工作责任制，深入开展结对帮扶工作，努力使更多贫困人口脱贫，早日过上小康生活。

千方百计扩大就业创业。大力实施就业援助帮扶活动，加强高校毕业生、农村转移劳动力和城镇困难人员就业工作。扎实做好农民工工作，加大技能培训力度，引导外出务工人员回流创业带动产业转移。新增农村劳动力转移就业3.8万人以上。

不断完善社会保障体系。健全城乡居民基本养老保险、城镇职工养老保险制度，推进失地农民参加养老保险工作，实现城乡养老全覆盖。积极落实机关事业单位养老保险制度改革措施，同步推进工资制度改革。实施全民参保登记计划，积极推进“五险合一”，力争实现全市社会保障“一卡通”。推动社会救助机制建设，完善城乡低保标准和农村“五保”供养标准自然增长机制，不断提高对弱势群体的救助水平。进一步加大社会保障力度，筹措城乡居民养老保险财政补助资金2.5亿元；城乡居民医疗保险财政补助标准提高到360元，努力消除群众生活后顾之忧。

大力发展卫生计生文体事业。推进县级公立医院改革，扩大基本药物制度实施范围，改革医保支付方式，推进市级新农合统筹和大病保险工作，加快困难群体医疗救助制度建设。继续完善医疗卫生服务体系，进一步完善诚信计生长效机制。逐步完善公共文化服务体系，继续推行文化惠民政策，抓好文艺精品创作，确保市文化艺术中心上半年向市民开放，确保完成100个新农村电影院建设。广泛开展全民健身运动，推进完善四级基本公共体育设施建设。加强档案管理工作。

加强和创新社会治理。健全打防管控一体化运作的立体化社会治安防控体系，全面加强反恐工作，提升社会公众安全感和满意度。深化基层平安建设，推行网格化服务管理，加快信息化建设步伐。完善党政军警民联合管边控边工作体系，提升边境防控水平。进一步加强法制宣传教育。推进信访工作制度改革，建立健全利益表达、协商沟通、救济救助等机制。加强突发事件应急处置工作，建设政府应急综合指挥平台。强化安全隐患排查整治，有效防范和坚决遏制重特大事故发生。强化食品、药品、农产品监管，保障质量安全。

实施为民办实事工程。筹措资金40亿元继续实施10项为民办实事工程，重点抓好农村公路、农村安全饮水、农户住房保险、城镇保障性住房、幼儿园、绿化示范村屯等六个方面的民生项目建设。

加强人才工作。坚持人才培养与引进并举，围绕支柱产业、特色产业、新产业的发展需要，广泛吸引各类高层次人才。继续加强人才小高地建设。

加强和改进新形势下民族工作。深入开展民族团结进步创建活动，打牢民族团结进步事业基础。加强宗教工作。

支持国防和军队建设。加强国防动员和国防后备力量建设，抓好人民防空工作，深入开展全国双拥模范城创建活动，推动军民深度融合发展。

三、推进依法行政，建设法治政府

面对新常态新形势新要求，必须坚持全面依法治国的基本方略，加快建设法治政府。

（一）加快职能转变。进一步规范政府部门的职责权限，积极推行权力清单、责任清单和负面清单管理制度，不断提高行政效能和服务水平。严格机构编制管理，确保财政供养人员只减不增。统筹整合乡镇资源，深化乡镇机构改革。

（二）依法履行职能。坚持以“法定职责必须为，法无授权不可为”作为政府行使权力的准则，坚决纠正不作为、乱作为，坚决惩处失职、渎职。加强和改进规范性文件清理、制定及监督管理工作。

（三）强化依法决策。坚持开门纳谏、广开言路，建立健全决策咨询制度，确保决策制度科学、程序正当、过程公开、责任明确。加强政务公开，推进政社分开、政事分开和行政权力透明运行。

（四）严格依法执法。加大关系群众切身利益的重点领域执法力度，落实行政执法责任制和过错责任追究制，规范执法行为。健全行政复议案件审理机制，纠正违法或不当行政行为，做到严格规范公正文明执法。

（五）自觉接受监督。强化决策执行监督，自觉接受人大及其常委会监督和人民政协民主监督，认真听取各民主党派、工商联、无党派人士、人民团体和社会各界人士的意见。充分发挥行政监察、审计监督等专门机构的作用，高度重视人民群众监督和新闻舆论监督。畅通监督渠道，健全网上受理、网下办理、网上回复的工作机制。

（六）加强作风建设。按照全面从严治党的要求，巩固拓展教育实践活动成果，推进政府作风建设。自觉践行“三严三实”要求，推行“145”领导干部工作法，坚决反对和纠正“四风”。严格落实党风廉政建设责任制，切实履行“一岗双责”，深入推进反腐倡廉。完成全市行政机关和参公单位公务用车制度改革。严格遵守中央“八项规定”，“三公”经费、会议费、培训费等一般性支出实行“零增长”，将财政资金更多投向民生保障和公共服务领域，让人民群众共享改革发展成果。

各位代表！新常态引领新发展，新使命开启新征程。让我们紧密团结在以习近平同志为总书记的党中央周围，在市委的坚强领导下，适应新常态、主动多作为，提振精气神、汇聚正能量，锐意进取、扎实工作，为全面完成今年和“十二五”目标任务，加快实现“两加”“两成”而努力奋斗！
